# Abrogation of FGFR signaling blocks **β**-catenin–induced adrenocortical hyperplasia and aldosterone production

**DOI:** 10.1172/jci.insight.184863

**Published:** 2025-09-16

**Authors:** Vasileios Chortis, Dulanjalee Kariyawasam, Mesut Berber, Nick A. Guagliardo, Sining Leng, Betul Haykir, Claudio Ribeiro, Manasvi S. Shah, Emanuele Pignatti, Brenna Jorgensen, Lindsey Gaston, Paula Q. Barrett, Diana L. Carlone, Kleiton Silva Borges, David T. Breault

**Affiliations:** 1 Division of Endocrinology, Boston Children’s Hospital, Boston, Massachusetts, USA.; 2 Department of Pediatrics, Harvard Medical School, Boston, Massachusetts, USA.; 3 Department of Pharmacology, University of Virginia, Charlottesville, Virginia, USA.; 4 Harvard Stem Cell Institute, Cambridge, Massachusetts, USA.

**Keywords:** Cardiology, Endocrinology, Homeostasis, Hypertension, Mouse models

## Abstract

Fibroblast growth factor receptors (FGFRs) are tyrosine kinase receptors critical for organogenesis and tissue maintenance, including in the adrenal gland. Here we delineate the role of FGFR2 in the morphogenesis, maintenance, and function of the adrenal cortex with a focus on the zona glomerulosa (zG). zG-specific *Fgfr2* deletion (Fgfr2-cKO) resulted in impaired zG cell identity, proliferation, and transdifferentiation into zona fasciculata (zF) cells during postnatal development. In adult mice, induced deletion of *Fgfr2* led to loss of mature zG cell identity, highlighting the importance of FGFR2 for the maintenance of a differentiated zG state. Strikingly, Fgfr2-cKO was sufficient to fully abrogate β-catenin–induced zG hyperplasia and to reduce aldosterone levels. Finally, short-term treatment with pan-FGFR small molecule inhibitors suppressed aldosterone production in both WT and β-catenin gain-of-function mice. These results demonstrate a critical role for FGFR signaling in adrenal morphogenesis, maintenance, and function and suggest that targeting FGFR signaling may benefit patients with aldosterone excess and/or adrenal hyperplasia.

## Abstract 2

This work demonstrates that Fibroblast Growth Factor Receptor 2 is critically involved in postnatal adrenal morphogenesis, and abrogation of FGFR signaling blocks β-Catenin-induced adrenocortical hyperplasia and aldosterone production.

## Introduction

Aldosterone is the main mineralocorticoid produced in the outermost zone of the adrenal cortex (zona glomerulosa [zG]) and acts as a critical regulator of circulating blood volume and Na^+^/K^+^ balance through activation of the mineralocorticoid receptor in the renal collecting tubules (1). Impaired development and maintenance of the zG mediates significant pathology through excessive or insufficient mineralocorticoid secretion. For example, mineralocorticoid deficiency (hypoaldosteronism) is associated with hyperkalemia and metabolic acidosis in adults and can result in life-threatening volume depletion through urine salt wasting in children (2). On the other hand, mineralocorticoid excess (primary aldosteronism [PA]) is the most common endocrine cause of hypertension and hypokalemia and is associated with significant cardiovascular morbidity and mortality (3, 4). Thus, a better understanding of the mechanisms that mediate zG morphogenesis, homeostasis, and aldosterone production is of major clinical relevance.

We previously demonstrated that zG cells give rise to adjacent glucocorticoid-producing zona fasciculata (zF) cells through a process of transdifferentiation (5). This process is blocked in zG-specific β-Catenin gain-of-function (βCat-GOF) transgenic mice, resulting in progressive zG hyperplasia and increased aldosterone production (6). Notably, GOF mutations in *CTNNB1* (the gene encoding β-catenin) have been implicated as driver mutations in patients with PA (7–10) and constitutive activation of the Wnt/βCat pathway is a common feature of aldosterone-producing adenomas (11). Analysis of βCat-GOF adrenal glands revealed prominent changes in genes controlling epithelial morphogenesis, including an upregulation of *Fgfr2*, the gene encoding the transmembrane tyrosine kinase receptor fibroblast growth factor receptor 2 (FGFR2), and its downstream target *Shroom3* (12).

FGFR2 belongs to the family of tyrosine kinase receptors (FGFRs 1–4) that mediate FGF signaling. This signaling pathway plays essential roles in early embryogenesis and organogenesis, and it remains important in adult life where it is critically involved in tissue maintenance and in the injury response of several organs, including the heart and lungs (13). FGFR2 is involved in the expansion of the adrenal primordium in fetal life; global deletion of *Fgfr2* results in hypoplasia of several organs, including the adrenal gland, and *Fgfr2*-null embryos die at birth (14, 15). Precisely how FGFR2 regulates postnatal adrenal development and zG cell maintenance remains unexplored. Our recent work showed that zG-specific conditional KO of *Fgfr2* leads to the development of a hypoplastic and disorganized zG in postnatal life and to a state of compensated hypoaldosteronism (12). These results indicate that FGFR2 is a key regulator of postnatal zG morphogenesis and maintenance and, combined with the previous observations, may be critically involved in the pathogenesis of βCat–driven zG hyperplasia and PA.

Here, we show that FGFR2 loss during postnatal adrenal morphogenesis disrupts their ability to proliferate, maintain their cellular identity, transdifferentiate into zF cells, and survive. In addition, FGFR2 is critical for maintenance of mature zG cell identity in the adult. Importantly, FGFR2 is required for the development of zG hyperplasia resulting from βCat-GOF. Finally, pharmacological inhibition of FGFR signaling lowers circulating aldosterone levels and impedes zG cell proliferation, which may provide a new treatment opportunity for patients with PA.

## Results

### FGFR2 maintains zG cell identity and proliferation during postnatal morphogenesis.

**Figure 1:**
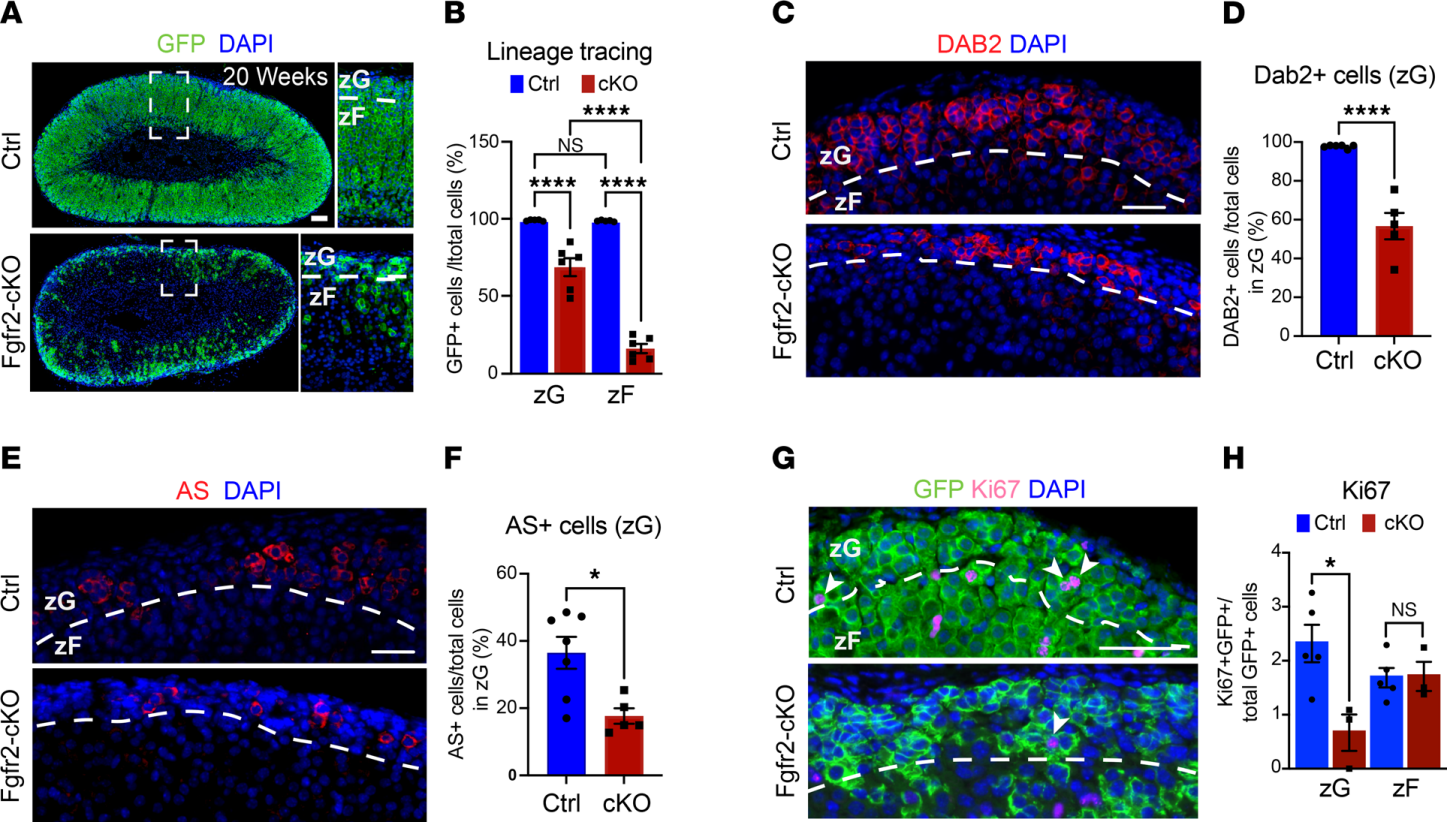
Tissue-specific *Fgfr2* KO (Fgfr2 cKO) impairs zG cell identity and transdifferentiation. (**A**) Lineage-tracing by immunofluorescence for GFP^+^ cells in adrenal glands from 20-week-old female Fgfr2-cKO and control mice (Ctrl, *AS^Cre/+^* *R26R^mTmG/+^*). White dashed box demarcates region enlarged to the right. (**B**) Quantification of GFP^+^ cells within the zG and zF from female Ctrl and Fgfr2-cKO (cKO) mice (20–24 weeks). One-way ANOVA with post hoc Tukey’s test, ****P < 0.0001, *n* = 5, 6 mice, respectively. (**C**) Representative images of DAB2 immunostaining in adrenal glands from adult female Ctrl and Fgfr2-cKO mice (10–24 weeks). (**D**) Quantification of DAB2^+^ cells in female Ctrl and Fgfr2-cKO mice (10–24 weeks). Student’s *t* test, ****P < 0.0001, *n* = 6, 5, respectively. (**E**) Representative pictures of Aldosterone Synthase (AS; Cyp11b2) immunostaining in adrenal glands from female Ctrl and Fgfr2-cKO mice (10–24 weeks). (**F**) Quantification of AS^+^ cells in female Ctrl and Fgfr2-cKO mice (10–24 weeks). Student’s *t* test, *P < 0.05, *n* = 7, 5, respectively. (**G**) Representative images of coimmunostaining of GFP (green) and Ki67 (magenta) in Ctrl and Fgfr2-cKO adrenal glands from female mice (20–24 weeks). White arrowheads point to GFP and Ki67–copositive cells. (**H**) Quantification of GFP and Ki67–copositive cells as a proportion of total GFP^+^ cells in the zG and zF of female Ctrl and Fgfr2-cKO mice (20–24 weeks). Student’s *t* test, *P < 0.05. *n* = 5, 3 mice, respectively. Scale bars: 50 μm. DAPI (blue), nuclei. Dashed white lines correspond to the zG-zF boundary.

To extend our earlier studies, and to determine the effect of FGFR2 loss on postnatal adrenocortical development, we used a zG-specific, conditional *Fgfr2*-KO mouse model combined with a lineage-tracing reporter allele (*AS^Cre/+^* *Fgfr2^fl/fl^* *R26R^mTmG/+^*, referred to as Fgfr2-cKO) (Supplemental Figure 1A; supplemental material available online with this article; https://doi.org/10.1172/jci.insight.184863DS1). In this model, Cre recombination occurs specifically in zG cells expressing *Cyp11b2*, the gene that encodes the steroidogenic enzyme aldosterone synthase (AS), which begins around the time of birth and is coincident with the start of postnatal zG morphogenesis (12). We have previously employed *AS^Cre/+^* *R26R^mTmG/+^* (control) mice to show that AS^+^ zG cells expressing membrane-targeted GFP migrate centripetally and transdifferentiate into zF cells during adrenocortical turnover (5). Consistent with this, we found that, in control mice, the AS^+^ lineage and its descendants (as indicated by GFP expression) occupy most of the zG and part of the zF by 6 weeks of age and populate nearly the entire cortex by 20 weeks of age (Figure 1, A and B, and Supplemental Figure 1, B and C). In contrast, in Fgfr2-cKO adrenal glands, lineage-tracing was severely disrupted. At 6–10 weeks of age, GFP^+^ cells occupied only approximately 40% of the zG (versus > 80% in controls) and 5% of the zF (versus 20% in control mice) (Supplemental Figure 1, B and C). By 20 weeks of age, approximately 70% of zG cells and < 20% of zF cells in Fgfr2-cKO adrenal glands were GFP^+^ compared with nearly 100% of both zG and zF cells in controls (Figure 1, A and B). The decrease in lineage-tracing in Fgfr2-cKO adrenal glands raised the possibility of a defect in zG cell identity. To address this, we assessed the effect of FGFR2 loss on markers of zG cell identify and observed a significant decrease in the proportion of cells that express the zG-specific markers disabled homolog 2 (DAB2) and AS (*Cyp11b2)*, hallmarks of a mature zG cell (16), which was similar in both sexes despite the fact that *Fgfr2* was expressed at ~2-fold higher levels in male mice (Figure 1, C–F, and Supplemental Figure 1, D–H). We also confirmed that the phenotype of perturbed lineage-tracing and zG cell identity persists in aged animals (18–20 months) (Supplemental Figure 2A). We next asked whether FGFR2 loss also influenced zG cell proliferation using coimmunostaining for the proliferation marker Ki67 and GFP. We found that the fraction of Ki67/GFP-copositive cells within the zG was reduced by ~4-fold in female Fgfr2-cKO adrenal glands compared with controls (Figure 1, G and H), indicating that FGFR2 loss impedes zG cell proliferation; conversely, proliferation of GFP^+^ cells in the zF was not affected.

The fact that the zG/zF ratio of the percent of GFP^+^ cells was higher in Fgfr2-cKO animals in comparison with controls (e.g., 8 versus 4 at 6–10 weeks of age; Supplemental Figure 1C) suggested a defect in zG to zF cell transdifferentiation in Fgfr2-cKO animals. On the other hand, loss of FGFR2 leading to an increase in cell death might also explain this finding. To explore this possibility, we performed immunostaining for activated caspase 3 and assessed apoptosis in the adrenal cortex of control and Fgfr2-cKO mice compared with dexamethasone-treated control mice (used as a positive control for apoptosis) (Supplemental Figure 2, B–D). While the level of apoptosis was markedly lower in control and Fgfr2-cKO mice than in dexamethasone-treated mice, we did observe a significant, albeit modest, increase in apoptosis in both the zG and in the outer zF of Fgfr2-cKO adrenal glands, which may partially contribute to the decline in GFP^+^ cells in the zF of these animals.

To explore the effect of FGFR2 loss on the zF, which comprises the majority of the cortex, we assessed zF thickness and morphology in Fgfr2-cKO and control adrenal glands using immunostaining for the zF-specific steroidogenic enzyme CYP11B1 (a mature zF cell marker essential for glucocorticoid synthesis). zF thickness and morphology were not affected by Fgfr2-cKO (Supplemental Figure 3, A and B), despite the decreased presence of GFP^+^ cells in the zF of Fgfr2-cKO adrenal glands; this may be explained by activation of the “alternative” pathway for zF renewal, as previously shown (5). Wilms tumor suppressor gene 1 (*Wt1*) expression was not increased, suggesting that compensatory activation of WT1^+^ capsular cells is unlikely to account for this phenotype (Supplemental Figure 3C). Consistent with this preservation of zF morphology, male Fgfr2-cKO animals had normal circulating corticosterone levels (Supplemental Figure 3D). Moreover, we measured adrenal weight in Fgfr2-cKO and control male mice, which revealed no change with FGFR2 loss, likely due to the relatively small contribution of the zG to the total adrenal mass and the ability of FGFR2-deficient adrenal glands to maintain a morphologically normal zF (Supplemental Figure 3E).

Together, these data show that FGFR2 is important for postnatal zG morphogenesis and that its loss results in disruption of zG cell identity and an increase in cell death, which likely both affect the ability of zG cells to undergo transdifferentiation. This extends our previous findings that Fgfr2-cKO mice exhibit decreased zG rosette frequency and glomerular area, along with compensated hypoaldosteronism (elevated renin in the setting of inappropriately normal aldosterone levels) (12).

### FGFR2 is important for maintenance of zG cell identity in the adult.

**Figure 2:**
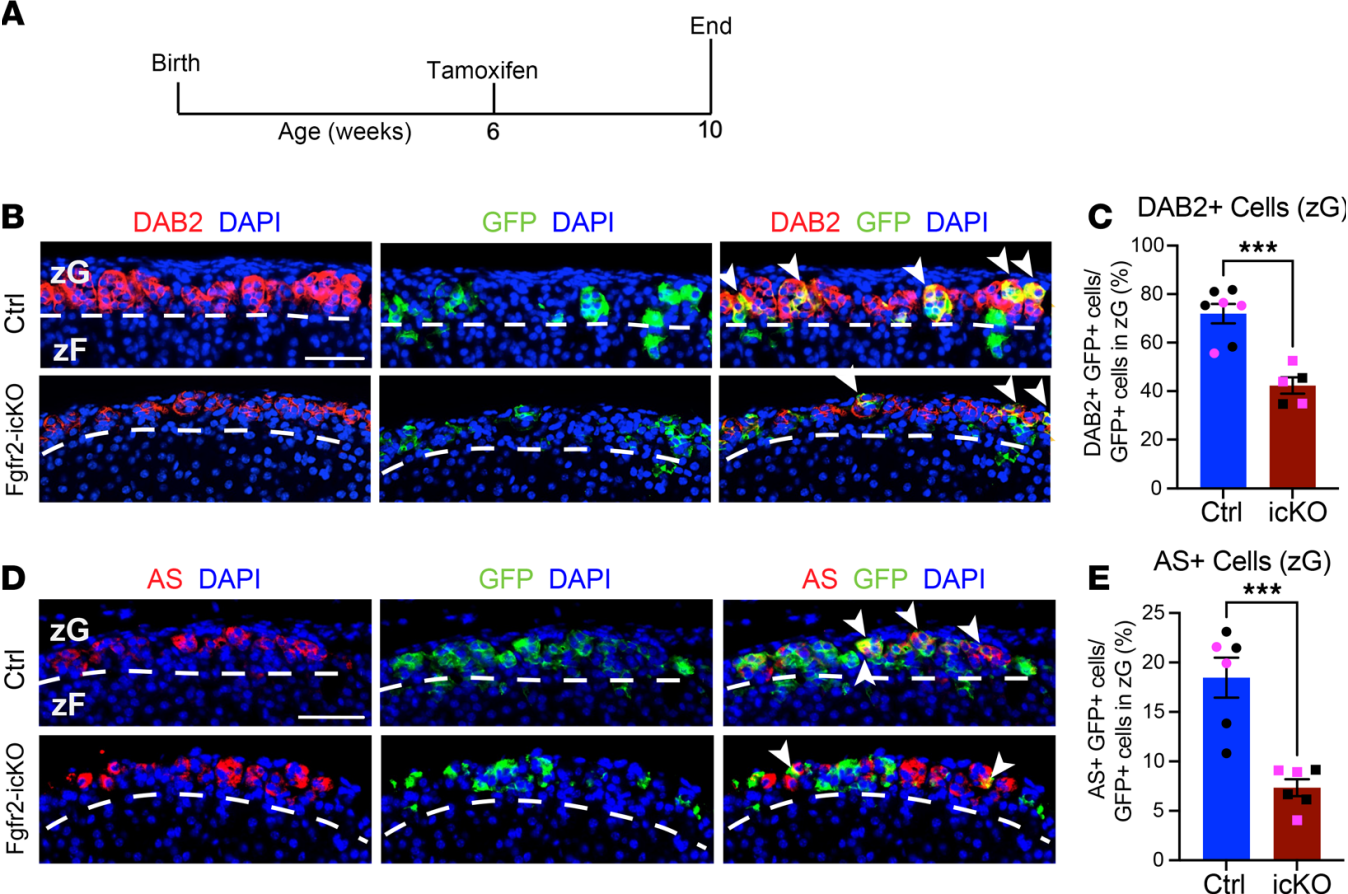
Inducible KO of *Fgfr2* (Fgfr2-icKO) in adults leads to a loss of zG cell identity. (**A**) Schematic of *AS^CreER^* induction with tamoxifen. (**B**) Representative images of GFP and DAB2 coimmunostaining in adrenal glands from adult *AS^CreER/+^* *R26R^mTmG/+^* (Ctrl) and *AS^CreER/+^ Fgfr2^fl/fl^ R26R^mTmG/+^* (Fgfr2-icKO) mice at 10 weeks of age (4 weeks after tamoxifen). White arrowheads point to GFP and DAB2–copositive cells. (**C**) Quantification of DAB2 and GFP–copositive cells as a proportion of total GFP^+^ zG cells in male and female Ctrl and Fgfr2-icKO (icKO) mice. Adrenal glands from male mice are represented with black dots; adrenal glands from female mice are represented with magenta dots. Student’s *t* test, ***P < 0.001, *n* = 7, 5, respectively. (**D**) Representative images of GFP and AS coimmunostaining in adrenal glands from adult Ctrl and Fgfr2-icKO mice at 10 weeks of age (4 weeks post tamoxifen). White arrowheads point to GFP and AS–copositive cells. (**E**) Quantification of GFP and AS–copositive cells as a proportion of total GFP^+^ zG cells in male and female Ctrl and Fgfr2-icKO mice. Adrenal glands from male mice are represented with black dots; adrenal glands from female mice are represented with magenta dots. Student’s *t* test, ***P < 0.001, *n* = 6, 6, respectively. Scale bars: 50 μm. DAPI (blue), nuclei. Dashed white lines correspond to the zG-zF boundary.

To establish whether FGFR2 is necessary for maintenance of a mature zG cell identity in the adult, we first developed a tamoxifen-inducible, zG-specific Cre recombinase mouse model (*AS^CreER/+^*) by retargeting the *AS^Cre/+^* allele in embryonic stem (ES) cells (Supplemental Figure 4, A and B). To validate expression of Cre recombinase in this model, we generated *AS^CreER/+^* *R26R^mTmG/+^* mice (Supplemental Figure 4C) and assessed GFP expression (Cre reporter activity) 1 week following tamoxifen treatment, and this revealed zG-specific expression of GFP (Supplemental Figure 4, D and E). At 4 months after tamoxifen treatment, we observed a progression of GFP^+^ cells from the outer to the inner zF in adrenal glands from both sexes, in keeping with the model of centripetal migration and transdifferentiation of zG into zF cells (Supplemental Figure 4E). Next, to assess the role of FGFR2 in the adult, we generated Fgfr2-icKO mice (*AS^CreER/+^* *Fgfr2^fl/fl^* *R26R^mTmG/+^*) (Supplemental Figure 4F). To evaluate changes in zG cell identity following loss of FGFR2, we performed coimmunostaining for GFP and the zG markers DAB2 or AS one month following tamoxifen treatment in male and female mice (Figure 2A). We observed only ~42% of GFP^+^ cells in Fgfr2-icKO adrenal glands costaining for DAB2 compared with > 75% in controls (Figure 2, B and C) and ~7% costaining for AS compared with ~18% in controls (Figure 2, D and E). These findings establish that FGFR2 is important for the maintenance of mature zG cell identity in the adult.

### FGFR2 loss abrogates βCat–induced zG hyperplasia.

**Figure 3:**
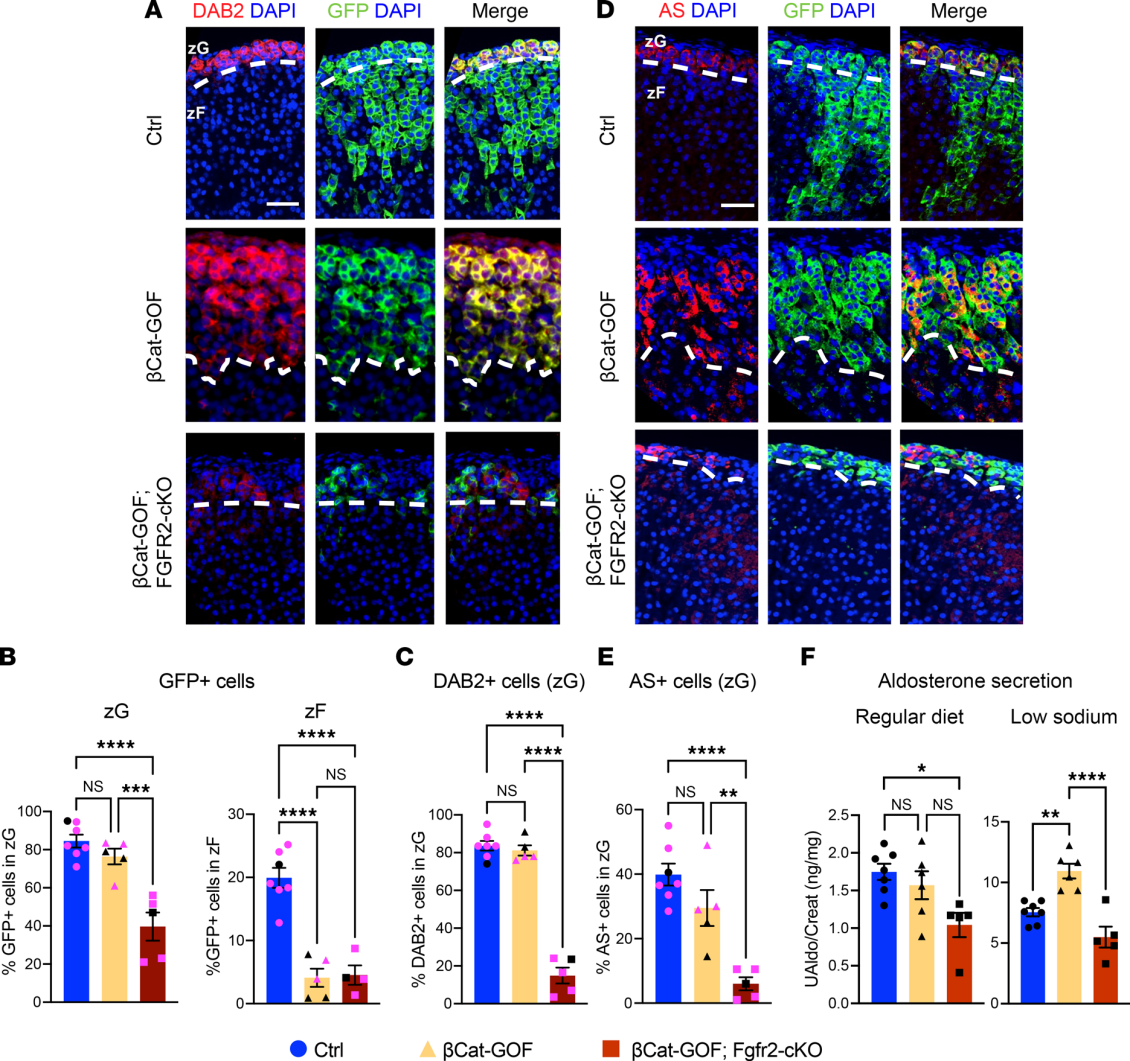
*Fgfr2* deletion abrogates β-catenin–induced zG hyperplasia. (**A**) Representative images of GFP (green) and DAB2 (red) immunostaining of Ctrl (*AS^Cre/+^* *R26R^mTmG/+^*), βCat-GOF, and βCat-GOF Fgfr2-cKO adrenal glands from adult mice (6–10 weeks). (**B**) Quantification of GFP^+^ cells as a proportion of total cells in the zG (left) and the zF (right) in adult male and female mice (6–10 weeks). One-way ANOVA, ***P < 0.001, ****P < 0.0001, *n* = 5–7 per group. (**C**) Quantification of DAB2^+^ cells in the zG of adult male and female mice (6–10 weeks). One-way ANOVA with post hoc Tukey’s test, ****P < 0.0001, *n* = 5–7 per group. (**D**) Representative images of GFP (green) and AS (red) immunostaining of Ctrl, βCat-GOF, and βCat-GOF Fgfr2-cKO adrenal glands from adult mice (6–10 weeks). (**E**) Quantification of AS^+^ cells in the zG of adult male and female mice. One-way ANOVA with post hoc Tukey’s test, **P < 0.01, ****P < 0.0001, *n* = 5–7 per group. Adrenal glands from male mice are represented with black dots; adrenal glands from female mice are represented with magenta dots (**B**, **C**, and **E**). (**F**) Mean 24-hour urine aldosterone corrected for creatinine in male Ctrl, βCat-GOF, and βCat-GOF Fgfr2-cKO mice (6–20 weeks). Mice were fed with normal chow for 4 days, followed by low-sodium chow for 6 days. Urine was collected daily for days 1–4 (regular diet, normal sodium) and 8–11 (low sodium). One-way ANOVA with post hoc Tukey’s test, *P < 0.05, **P < 0.01, ****P < 0.0001, *n* = 5–7 per group. Scale bars: 50 μm. DAPI (blue), nuclei. Dashed white lines correspond to the zG-zF boundary.

We have previously shown that conditional activation of WNT/βCat signaling in the zG, following stabilization of βCat-GOF, leads to zG hyperplasia due to a block in transdifferentiation of zG to zF cells, together with persistence of zG cell identity (6). Notably, *Fgfr2* was among the most upregulated genes in the adrenal glands of these mice (12). To test whether FGFR2 is required for the development and/or maintenance of βCat–driven zG hyperplasia, we generated βCat-GOF Fgfr2-cKO bigenic mice (*AS^Cre/+^* *Ctnnb1^fl(ex3)/+^* *Fgfr2^fl/fl^* *R26R^mTmG/+^*) and βCat-GOF monogenic mice (*AS^Cre/+^* *Ctnnb1^fl(ex3)/+^* *R26R^mTmG/+^*) (Supplemental Figure 5A) and assessed lineage-tracing, zG cell differentiation markers, proliferation, and aldosterone production. Remarkably, we found that *Fgfr2* deletion was sufficient to fully abrogate βCat–induced zG hyperplasia in bigenic mice from both sexes (Figure 3, A–E). The proportion of GFP^+^ cells in the zG of bigenic mice was more than 50% reduced compared with βCat-GOF mice (and controls), recapitulating the phenotype of Fgfr2-cKO animals (Figure 1 and Supplemental Figure 1). We also observed a 4-fold decrease in the proportion of GFP^+^ cells in the zF of bigenic mice compared with controls. We previously showed that the zG hyperplasia observed in βCat-GOF mice results from a block in transdifferentiation of zG to zF cells, rather than from an increase in proliferation (6). Consistent with this, we observed no difference in the proliferation rate of GFP^+^ cells from control, βCat-GOF, and βCat-GOF Fgfr2-cKO bigenic adrenal glands (Supplemental Figure 5, B and C), suggesting that in bigenic mice, the absence of zG hyperplasia cannot be ascribed to a direct effect on proliferation. These findings indicate that Fgfr2-cKO is dominant over βCat-GOF and is sufficient to prevent the development of zG hyperplasia, likely due to the defect in zG cell identity that prevents them from responding to the effects of βCat-GOF.

Next, to assess the effect of these genetic modifications on zG cell function, we measured 24-hour urinary aldosterone secretion in control, βCat-GOF, and βCat-GOF Fgfr2-cKO male mice. In this cohort of relatively young adult mice on a regular sodium diet, urine aldosterone levels were similar between control and βCat-GOF mice, despite the underlying zG hyperplasia (Figure 3F), likely because aldosterone excess in this model is progressive with age and less pronounced in young animals (6, 12). Compared with control mice, βCat-GOF Fgfr2-cKO mice showed a modest but significant decrease in aldosterone secretion on a regular sodium diet. Notably, βCat-GOF mice were hypersensitive to activation of the renin-angiotensin-aldosterone system (RAAS) induced by a low sodium diet, resulting in an approximately 1.6-fold increase in urinary aldosterone output compared with control mice, which was restored to control levels in βCat-GOF Fgfr2-cKO mice (Figure 3F). Together, these results demonstrate the capacity of *Fgfr2* deletion in zG cells to fully prevent the development of βCat–driven adrenal hyperplasia and to block aldosterone excess following activation of RAAS.

### Fgfr2-cKO adrenal glands have fewer Ang II–responsive cells.

We previously showed that Fgfr2-cKO mice show an increase in plasma renin activity, consistent with an important role for FGFR2 in maintaining normal levels of aldosterone production (12). It is also known that FGFR signaling can increase intracellular Ca^2+^ activity through phosphorylation of phospholipase C (PLC) (13). Given the well-established role of Ca^2+^ signaling as a critical second messenger mediating aldosterone production (17), we sought to determine if *Fgfr2* deletion alters the zG Ca^2+^ response to angiotensin II (Ang II), a potent aldosterone secretagogue that elicits robust Ca^2+^ oscillations with stereotypic bursting behavior in ex vivo adrenal slices (18). To test this, we generated *Fgfr2-cKO* *ROSA26^floxGCaMP6/HZE^* and control (*AS^Cre/+^* *ROSA26^floxGCaMP6/HZE^*) mice that express the genetically encoded Ca^2+^ indicator GCaMP6 and measured changes in GCaMP6 fluorescence intensity in response to 300 pM Ang II (18). In Fgfr2-cKO*^GCaMP6^* slices, we found a marked decrease in the number of zG cells with oscillatory Ca^2+^ activity activated by Ang II compared with control adrenal slices (Supplemental Figure 6A), consistent with an overall decrease in the number of mature zG cells in Fgfr2-cKO adrenal glands (Figure 1 and Supplemental Figure 1), and possibly a defect in rosette formation and/or adherens junctions (12, 18). By contrast, analysis of Ca^2+^ oscillations (events) among Ang II–responsive cells (mean events per cell, mean event period, and mean number of events/burst) revealed no differences between Fgfr2-cKO and control adrenal glands (Supplemental Figure 6, B–D). These results suggest that Ca^2+^ signaling, and the pathways leading to its activation (e.g., Ang II), are not directly affected by FGFR2 loss and that the decreased number of cells actively showing Ca^2+^ oscillations is explained by the decrease in mature zG cells in Fgfr2-cKO adrenal glands.

### Pharmacological inhibition of FGFR signaling blunts aldosterone production and impedes zG cell proliferation.

**Figure 4:**
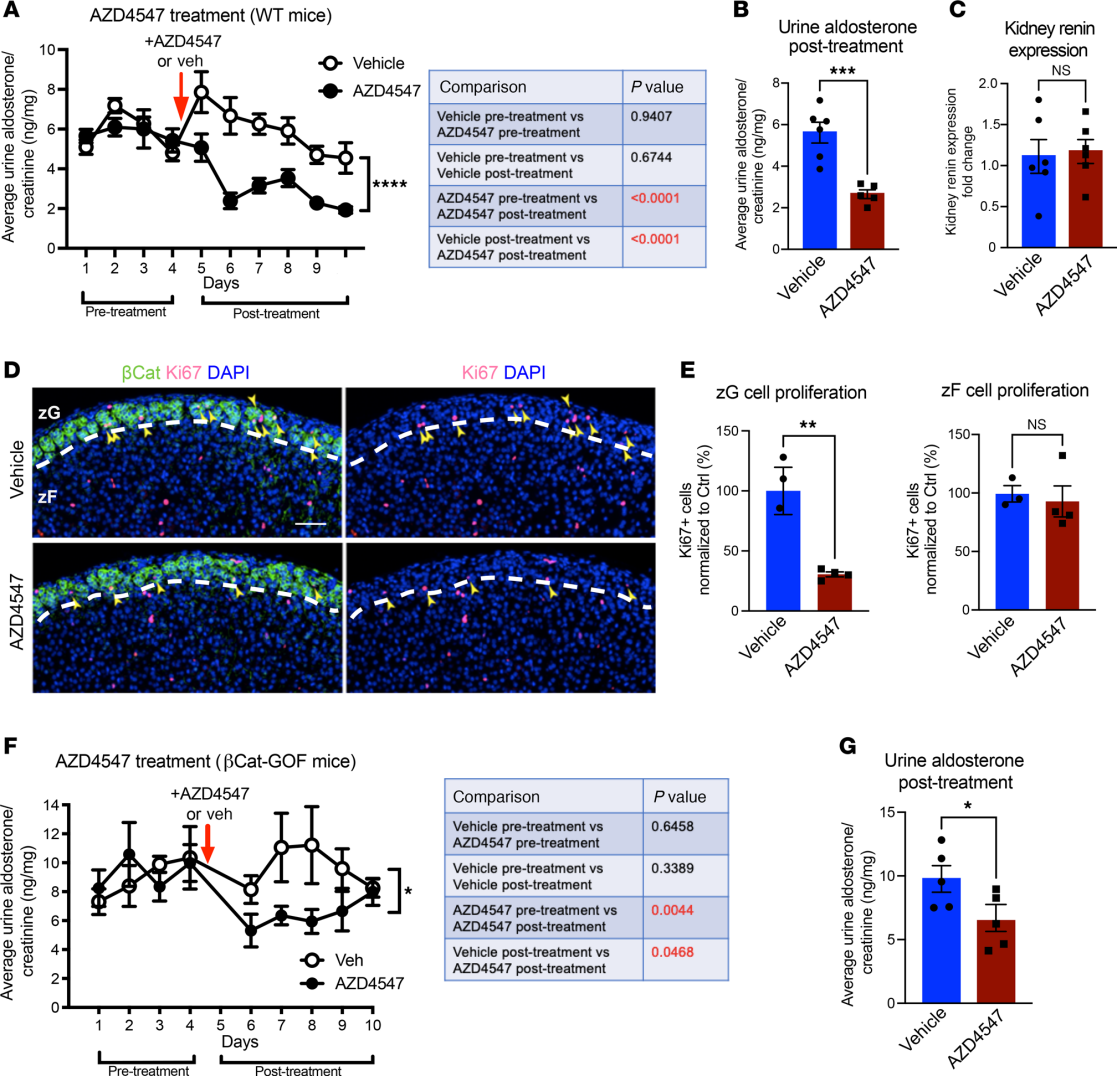
Pharmacological inhibition of FGFR lowers aldosterone secretion and zG proliferation. (**A**) Urinary aldosterone excretion (corrected for creatinine) of adult male C57BL/6J (WT) mice (12 weeks) treated with 30 mg/kg/day AZD4547 or vehicle per os starting on day 4 (red arrow). Two-way ANOVA followed by Fisher’s least significant difference test, ****P < 0.0001 (for comparison of posttreatment AZD4547 versus vehicle), *n* = 6, 5 mice, respectively. *P* values for all comparisons are shown in the table. Pretreatment: days 1–4; posttreatment: days 6–10. (**B**) Average urine aldosterone excretion (corrected for creatinine) from **A** starting 48 hours after AZD4547 (days 6–10). Student’s *t* test, *n* = 6, 5 mice, respectively. (**C**) Renin mRNA expression levels (qPCR) in kidneys from male C57BL/6J mice (7–8 weeks) treated with 10 mg/kg/day AZD4547 or vehicle by oral gavage for 7 days. Student’s *t* test, *n* = 6, 6 mice, respectively. (**D** and **E**) Representative images of Ki67 and β-Catenin immunostaining (**D**) and quantifications (**E**) in adrenal glands from male C57BL/6J mice (7–8 weeks) treated with 10 mg/kg/day AZD4547 or vehicle. Yellow arrowheads indicate Ki67^+^ zG cells. Student’s *t* test, *n* = 3, 4 mice, respectively. Scale bars: 50 μm. DAPI (blue), nuclei. Dashed white lines correspond to the zG-zF boundary. (**F**) Urinary aldosterone excretion (corrected for creatinine) of adult female βCat-GOF mice (36–39 weeks) treated with 30 mg/kg/day AZD4547 or vehicle per os starting on day 4 (red arrow). Two-way ANOVA followed by Fisher’s least significant difference test, *P < 0.05 (for comparison of posttreatment AZD4547 versus vehicle), *n* = 5, 5 mice, respectively. *P* values for all comparisons are shown in the table. Pretreatment: days 1–4; posttreatment: days 6–10. (**G**) Average urine aldosterone excretion (corrected for creatinine) from **F**, starting 48 hours after AZD4547 (days 6–10). Student’s *t* test, *n* = 5, 5 mice, respectively. All comparisons: *P < 0.05, **P < 0.01, ***P < 0.001, ****P < 0.0001.

Given that FGFR2 loss leads to a decrease in zG cell proliferation, loss of cellular identity, and hyperreninemic hypoaldosteronism in vivo (12), we next asked whether pharmacological inhibition of FGFR signaling could elicit similar effects. To address this, we selected a second-generation tyrosine kinase inhibitor (AZD4547) with high specificity against FGFR1/2/3 and minimal off-target effects on vascular endothelial growth factor receptors (VEGFR) (19). Indeed, treatment of WT C57BL/6J male mice with AZD4547 resulted in sustained suppression of urinary aldosterone output within 48 hours (Figure 4, A and B), while kidney renin expression was not significantly different (Figure 4C). Histological analysis also revealed a > 3-fold decrease in zG cell proliferation (Figure 4, D and E), recapitulating our findings from the Fgfr2-cKO model. Of note, this effect on cell proliferation was also zone specific, sparing zF cells. In addition, we observed a significant decrease in the expression of *Cyp11b2*, without a change in the zF marker *Cyp11b1* (Supplemental Figure 7A) as well as decreased expression of downstream FGFR targets through the Ras/Raf-MEK-ERK (MAPK) pathway, *Etv5* and *Dusp6* (13) (Supplemental Figure 7B). Probing expression levels of other factors known to affect adrenocortical cell proliferation (*Shh*, *Igf2*, *Rspo3*) (20–22) also revealed a significant downregulation of *Shh* (encoding Sonic Hedgehog) (Supplemental Figure 7C). Assessment of adrenal glands from male mice treated with an alternative pan-FGFR inhibitor (BGJ398) (23) showed a similar suppression of zG cell proliferation, with no effect on zF cell proliferation (Supplemental Figure 7, D and E). Importantly, treatment of female βCat-GOF mice with AZD4547 also led to a significant inhibition of aldosterone production, providing proof-of-concept for the ability of FGFR targeting to inhibit aldosterone in the context of adrenal hyperplasia (Figure 4, F and G), albeit with a possible amelioration of the effect at the end of the 6-day treatment course. These intriguing findings suggest that pharmacological inhibition of FGFR signaling may represent a potentially new treatment strategy to reduce circulating aldosterone levels in patients with hyperaldosteronism.

## Discussion

FGFR2 is expressed in the outer cortex of mouse and human adrenal glands (15, 24) and FGFR2 loss during fetal life leads to adrenal hypoplasia (14, 15), but the precise role FGFR2 plays during postnatal adrenal morphogenesis and maintenance of mature zG cell identity in the adult remained poorly understood. Our work shows that FGFR2 is a key regulator of zG cell identity both during early postnatal development and in the adult, with significant implications for their ability to efficiently transdifferentiate into zF cells. Indeed, FGFR2 loss dramatically blunts the numbers of steroidogenically active (DAB2- and AS-expressing) and Ang II–responsive cells within the zG. AS is the terminal enzyme in the mineralocorticoid synthetic pathway and is essential for the production of aldosterone. DAB2 is an adapter protein for cargo selection in clathrin-mediated endocytosis, and its expression in the adrenal cortex is limited to the zG, where it is involved in AS expression and aldosterone secretion (16). The decrease in AS- and DAB2-expressing cells within the zG leads to a biochemical profile of hyperreninemic hypoaldosteronism (high renin levels with inappropriately normal aldosterone), in which the depleted population of steroidogenically active zG cells in Fgfr2-cKO mice collectively maintains sufficient aldosterone output due to activation of RAAS (12). Inappropriately normal aldosterone levels have been described in patients with isolated congenital hyperreninemic hypoaldosteronism due to mutations in the *CYP11B2* gene (encoding AS) (25), in families without linkage to *CYP11B2* (26), and recently due to mutations in the R-Spondin receptor *LGR4/GPR48* (27). Inactivating mutations of *FGFR2* in humans are known to cause the rare lacrimo-auricular-dento-digital (LADD) syndrome, with a constellation of aplastic lacrimal ducts, hearing loss, dysplastic teeth, and digital malformations (28). It is not known whether such patients also experience subclinical mineralocorticoid deficiency; our work suggests that assessing renin and aldosterone levels would be advisable in this patient group.

Loss of FGFR2 led to a clear decrease in zG cell proliferation and an apparent decrease in their ability to transdifferentiate into zF cells. Although the progeny of AS^Cre^-expressing cells eventually populate most of the zF in control animals, only a small proportion of zG cells in Fgfr2-cKO mice appear to give rise to zF cells. Interestingly, spatial analysis of apoptosis by activated Caspase 3 staining revealed increased apoptosis in the zG and in the outer zF of Fgfr2-cKO mice. Taken together, these findings suggest that the marked decrease in GFP^+^ zF cells in Fgfr2-cKO mice can be explained by an inherent defect in the ability of GFP^+^ zG cells to transdifferentiate, an increased susceptibility to apoptotic cell death while migrating through the outer zF, or both. Nevertheless, the zF of Fgfr2-cKO adrenal glands remained phenotypically normal as assessed by immunostaining for the zF-specific marker CYP11B1. Previous studies have shown that nonsteroidogenic, AS^–^ zG cells expressing *Shh* (the gene encoding Sonic Hedgehog) also have progenitor cell properties (20, 29), as does a population of Gli1^+^ cells within the adrenal capsule (20, 30). Indeed, our data suggest that there exists a distinct, AS^–^ progenitor cell pool that can be mobilized to give rise to a structurally normal zF if the canonical process of centripetal zG cell migration of AS^+^ zG cells is disrupted. Consistent with this, we previously showed that an AS^Cre^-independent “alternative” pathway exists following zG-specific deletion of *Nr5a1* (the gene encoding Steroidogenic Factor 1) (5).

To specifically assess FGFR2’s role in the maintenance of zG cell identity, we generated a mouse with zG-specific tamoxifen-inducible Cre recombinase expression under the control of the endogenous *AS* (*Cyp11b2*) locus. This model enables genetic manipulation of zG cells in adult mice, thereby avoiding the developmental changes that might arise using the original *AS^Cre/+^* mouse model, in which Cre expression is coincident with AS expression from around the time of birth. Our tamoxifen-inducible model shows that FGFR2 is critical for maintenance of zG cell identity in adults. While we cannot exclude the possibility that increased apoptosis in the zG is also present in Fgfr2-icKO adrenal glands (as seen in the Fgfr2-cKO model), this is unlikely to explain the observed phenotype, as (a) the absolute increase in apoptosis seen in Fgfr2-cKO was very modest, and (b) persistent GFP^+^ expression in cells that have lost DAB2/AS expression suggests that these cells are still viable and have dedifferentiated. The fact that a minority of GFP^+^ cells in Fgfr2-icKO mice are still able to express DAB2 and AS suggests that FGFR2-independent mechanisms may also contribute to maintenance of zG identity. It is also possible that the small number of GFP^+^ cells in Fgfr2-icKO mice could result from differential recombination efficiency of the *Fgfr2* and the *mTmG* alleles in these cells. The development of this tamoxifen-inducible *AS^CreER^* mouse model will enable the study of other pathways of interest to the field of adrenocortical biology, especially when inducible expression of Cre recombinase is warranted, as recently demonstrated (31, 32).

The canonical Wnt/βCat pathway plays a critical role in normal zG development and maintenance, driving zG cell differentiation and function (6, 11, 12, 21, 29, 33–36). βCat-GOF mutations have been implicated as driver mutations in cohorts of patients with PA (7–11), although precisely how they transform normal adrenal tissue remains elusive. We have previously shown that mice with βCat-GOF progressively develop zG hyperplasia, eventually leading to aldosterone excess (6). Strikingly, this phenotype is fully abrogated by *Fgfr2* deletion (βCat-GOF Fgfr2-cKO). The histological phenotype of bigenic mice closely recapitulates that of monogenic Fgfr2-cKO mice, including impaired zG cell identity and likely impaired transdifferentiation. Together, these results indicate that FGFR2 loss is dominant over βCat-GOF and is sufficient to prevent the development of zG hyperplasia. An important question worth addressing in future studies is whether inhibition of FGFR2 can reverse established zG hyperplasia and the role of cell death in this process.

We have previously shown that Fgfr2-cKO mice develop a state of compensated hyperreninemic hypoaldosteronism (high renin levels with inappropriately normal blood aldosterone levels) (12). In this work, we demonstrate that pharmacological inhibition of FGFR signaling lowers aldosterone levels in WT mice, validating our earlier findings. Additionally, we showed that inhibition of FGFR signaling was also able to lower aldosterone levels in our mouse model of βCat–driven adrenal hyperplasia (βCat-GOF) following genetic (*Fgfr2* deletion) and pharmacological approaches. While our cohort of young adult βCat-GOF mice demonstrated normal aldosterone levels at baseline, these mice overproduced aldosterone in response to Ang II stimulation elicited by a low-salt diet. Importantly, this hyperresponsiveness to Ang II was blocked in βCat-GOF Fgfr2-cKO mice. These results are consistent with the complete absence of zG hyperplasia in βCat-GOF Fgfr2-cKO mice. Pharmacological inhibition of FGFR signaling in an older (8-month-old) cohort of βCat-GOF mice also resulted in a swift suppression of aldosterone secretion. We cannot exclude the possibility that the apparent convergence of aldosterone secretion in the AZD4547 and vehicle-treated groups on the last treatment day reflects amelioration of the drug’s effect with time through compensatory mechanisms (aldosterone escape), akin to what is often seen with ACE inhibitors (37). It will be of interest to explore the effect of prolonged treatment with FGFR inhibitors in this model, in a time frame (e.g., weeks to months) that may allow for possible reversal of zG hyperplasia. Additional work in other mouse models of hyperaldosteronism is also required to fully explore the translational potential of FGFR targeting as a novel strategy to mitigate aldosterone excess in patients with PA, or in the setting of secondary (hyperreninemic) aldosteronism, such as in patients with heart failure or liver failure. It will also be of interest to assess whether the inhibition of zG cell proliferation with FGFR inhibitors can be recapitulated in preclinical models of adrenocortical carcinoma. Furthermore, and of translational importance, our findings suggest that iatrogenic hypoaldosteronism may be an unrecognized consequence of FGFR inhibitors, which could conceivably aggravate known side effects of this group of drugs (e.g., fatigue, diarrhea) in oncological patients (38).

We previously showed that genetic inactivation of FGFR2 in the adrenal during early postnatal life causes hyperreninemic hypoaldosteronism in adult mice, yet in this study, acute FGFR inhibition did not increase *Ren* mRNA in the kidney despite reduced aldosterone levels. Nevertheless, acute compensatory RAAS activation may have occurred through alternative mechanisms, including (a) posttranslational processing of (pro)renin to renin, (b) regulation of (pro)renin secretory vesicle release and/or activation, or (c) (pro)renin activation of the (pro)renin receptor (39). We cannot exclude the possibility that inhibition of paracrine FGFR signaling directly in the kidney by AZD4547 may have interfered with renin expression.

Although our studies reveal that FGF signaling is a key mediator of zG cell identity and aldosterone production, it is not fully clear how this effect is mediated on a molecular level. Activated FGFRs are known to signal through 4 major pathways, including PLC, MAPK, phosphatidylinositol-3 kinase/protein kinase B (PI3K/AKT), and signal transducer and activator of transcription (STAT) (13), all of which are operative in zG physiology. PLC, MAPK, and STAT signaling can mediate Ang II–induced steroidogenesis (40, 41), while upregulation of PI3K/AKT signaling has been described in aldosterone-producing adenomas (42). Our calcium imaging results indicate that FGFR2 is unlikely to act by direct stimulation of Ca^2+^ signaling through PLC in zG cells, as *Fgfr2* deletion did not affect Ang II–elicited Ca^2+^ activity among active cells in adrenal slices. The downregulation of *Etv5* and *Dusp6* in the adrenal glands of mice treated with the FGFR inhibitor AZD4547 suggests that the MAPK pathway may be an important downstream mechanism of adrenocortical FGFR, although further studies confirming this are necessary. Interestingly, we also observed downregulation of *Shh*, suggesting possible interplay between FGF signaling and Hedgehog signaling, an important signaling molecule in the zG (20).

In conclusion, our work provides important insights into postnatal adrenocortical development and maintenance, illustrating the significance of FGFR2 as a key mediator of normal zG cell identity and maintenance. Importantly, we also show that FGFR2 loss can fully prevent the development of Wnt/βCat–driven adrenal hyperplasia and that FGFR inhibitors can decrease aldosterone secretion. Taken together, our findings suggest that FGFR inhibition may be a promising treatment strategy for patients with primary or secondary aldosterone excess.

## Methods

### Sex as a biological variable.

Male and female transgenic animals were used in this study, as indicated in figure legends, and no major sex differences were observed. In experiments using animals from both sexes, colored data points have been used to indicate the sex of each animal. For the experiments using pharmacological treatment (FGFR inhibitors) to assess effects on aldosterone, single-sex cohorts were used to reduce within-group variability (male C57BL/6J mice and female βCat-GOF mice).

### Animal experiments.

Mouse strains used in this study were: C57BL/6J (Jackson Laboratory), *AS^Cre/+^* [*Cyp11b2tm1.1(cre)Brlt*; ref. 5], *Ctnnb1^fl(ex3)^* (*Ctnnb1tm1Mmt*; ref. 43), *Fgfr2flox* (*Fgfr2tm1Dor*; ref. 44), *R26R-mTmG* [*Gt(ROSA)26Sortm4(ACTB-tdTomato-EGFP)Luo*; ref. 45], and *B6;129S-Gt(ROSA)26Sortm95.1(CAG-GCaMP6f)Hze/J* (Jackson Laboratory, Strain #:024105). Additionally, we used the newly generated inducible Cre mouse strain *AS^CreER^* (generation described separately below). All transgenic animals were maintained on a mixed sv129-C57BL/6J genetic background. PCR was used for genotyping. Littermates and both male and female animals were studied whenever possible. When indicated, mice were fed normal-chow (0.32% Na^+^) or low-salt (0.05% Na^+^) chow (ScottPharma Solutions). FGFR inhibitors AZD4547 and BGJ893 were purchased from Selleck Chemicals. The drugs were administered for 7 or 12 days by oral gavage (to assess effects on gene expression by quantitative PCR [qPCR] and adrenal histology, respectively). Alternatively, mice received drug for 6 days via a peanut butter pellet (to avoid the stress of daily handling) in the metabolic cage experiments, as detailed below. Dexamethasone suppression was performed using C57BL/6 male mice 8–10 weeks of age that were treated with either water-soluble dexamethasone (Sigma-Aldrich, 2915) at a concentration of 3.6 mcg/mL (~20 mcg/day assuming an average water intake of 5.8 mL/day) or no-drug control via drinking water for 4 days. Mice were housed on a 12-hour/12-hour light-dark cycle (lights on at 7 a.m.) with ad libitum access to rodent chow and water.

### Generation of the AS^CreER^ mouse strain.

To generate tamoxifen-inducible *AS^CreER^* mice, we retargeted the original *AS^Cre^* allele in the ES cell clone used to generate *AS^Cre^* mice (Supplemental Figure 4A) (5). We employed homologous recombination to insert the tamoxifen-sensitive ERT2 cassette in frame at the end of the Cre sequence (Supplemental Figure 4B). Positive selection was made possible using an FRT-PuroΔTK-FRT selection cassette, and negative selection was made possible using a Diphtheria Toxin A (DTA) selection cassette. The 3′ homology arm contained a modified fragment of the *Cyp11b2* locus fused with 1kb of *Cre* coding sequence (used in the original targeting vector) (5). This fragment was subcloned into a modified pBluescript, in which the polylinker between KpnI and SaII sites was replaced with a PacI-SaII-KpnI-PacI sequence. The 3′ homology arm contained a fragment of the FRT-flanked neomycin resistance cassette (used in the original targeting vector) (5). A fragment containing the *ERT2* sequence along with the FRT-PuroΔTK-FRT selection cassette was inserted between the 2 homology arms. As with the original *AS^Cre^* construct, the entire coding sequence of the *AS* gene was rendered null, retaining only exon 1, intron 1, and the first portion of exon 2 in the *AS^CreER^* construct. All potential translation start sites (ATG) within exon 1 or 2 were also excluded from the construct or mutated. Following homologous recombination in ES cells, positive selection and PCR analysis of genomic DNA were used to screen properly targeted clones. ES cells were injected into blastocysts, and founder mice were crossed with mice expressing flippase (FLP) recombinase, as previously described (5), to remove the FRT-flanked PuroΔTK and neomycin resistance sequences (Supplemental Figure 4B).

*AS^Cre^* and *AS^CreER^* mice were genotyped using the following primers: Forward: 5**′**-GAGCTGGGGCCCATTTTCAGG-3**′**, Reverse 1: 5**′**-GCTCCAGGTGCATCCGACGG-3**′**, Reverse 2: 5**′**-AACTTGCACCATGCCGCCCA-3**′** (Tm = 65°C). PCR product size was 640 for *AS^Cre^* and *AS^CreER^* alleles and 525 bp for the WT allele.

### Tamoxifen induction in mice.

To induce Cre recombinase, 6-week-old *AS^CreER^* mice were treated with a single dose of Tamoxifen (Sigma) at 10 mg per 25 g body weight. Tamoxifen was diluted in corn oil with 10% ethanol and administered by oral gavage.

### Tissue preparation.

After dissection, adrenal glands were trimmed of surrounding fat tissue, rinsed in phosphate-buffered saline (PBS), and weighed. For immunofluorescence, adrenal glands were cut into halves with a surgical blade and fixed in 4% paraformaldehyde (PFA) at 4°C for 1 hour. For RNAscope in situ hybridization, intact adrenal glands were fixed in 4% PFA at room temperature overnight.

### Paraffin section immunofluorescence.

After fixation, adrenal glands were dehydrated in ethanol and xylene and embedded in paraffin blocks. Paraffin sections were cut at 5 μm thickness. Sections were rinsed in xylene, an ethanol gradient and then PBS. Antigen retrieval was performed in Tris-EDTA pH 9.0 (for Cyp11b1 and Cyp11b2) or 10 mM Sodium Citrate pH 6.0 (all others). Sections were blocked in 5% Normal Goat Serum (NGS) or 5% Normal Donkey Serum (NDS), 0.1% Tween-20 in PBS for 1-2 hours at room temperature. Primary antibodies were diluted 1:100–1:200 in 5% NGS or 5% NDS in PBS and incubated on sections at 4°C overnight. Slides were washed 3 times for 5 minutes in 0.1% Tween-20 in PBS or 1% BSA in PBS. Secondary antibodies were diluted 1:200 in PBS and incubated on sections at RT for 1-2 hours. For nuclear staining, DAPI (4’,6-diamidino-2-phenylindole) was added to the secondary antibody mixture at a final concentration of 1:1,000. Slides were rinsed 3 times in PBS and then mounted with Prolong Gold Antifade Mountant with DAPI (Invitrogen, #P36931). Before mounting, some sections were incubated in TrueBlack Lipofuscin Autofluorescence Quencher (Biotium, #23007) in 70% EtOH at RT for 3 minutes. Primary antibodies included: mouse anti-βCat (BD Biosciences, 610153), chicken anti-GFP (Aves Labs, GF-1020), mouse anti-Dab2 (BD Biosciences, 610464), rabbit anti-Dab2 (Cell Signaling, 12906), rabbit anti-Cyp11b2 and Cyp11b1-555 (kindly provided by Celso E. Gomez-Sanchez), rabbit anti-Ki67 (Cell Signaling Technology, 9129), rabbit anti-activated Caspase 3 (BD Biosciences, 559565), and rabbit anti-cleaved caspase-3 antibody (abcam, ab32042). The following secondary antibodies were used: Alexa Fluor 647–conjugated goat anti-rabbit IgG, Alexa Fluor 647–conjugated goat anti-mouse IgG, Alexa Fluor 488–conjugated goat anti-chicken IgG (Invitrogen), and donkey anti-rabbit IgG/647 (Invitrogen, A-31573). Images were acquired using a Nikon upright Eclipse 90i microscope and a Zeiss Axio Imager.Z2 microscope and adjusted for brightness and contrast in ImageJ. Quantification of Ki67-, AS-, and DAB2-positive cells was performed using 4 representative 20x areas from equatorial adrenal sections. zG and zF were defined morphologically to perform zone-specific cell quantifications. For activated Caspase 3 quantification, the zF was further subdivided into outer and inner zF using ImageJ (NIH). To define the border between outer and inner zF, the midpoint of the zF radius (extending from the medulla-zF border to the zF-zG border) was determined at several (at least 5) points. Activated Caspase 3^+^ cells were quantified for the whole of the zG, outer zF and inner zF, and normalized to corresponding area.

### RNA extraction and quantitative PCR.

Whole adrenal glands or kidneys were homogenized using TRI reagent to isolate RNA, and cDNA was synthesized as described previously (32). Gene expression was assessed by quantitative real-time PCR using TaqMan Universal PCR Master Mix (Applied Biosystems) and QuantStudio 6 Flex thermocycler (Applied Biosystems). *Hprt* or *Actb* were used as housekeeping genes, and data were expressed using either 2^–ΔΔCt^ or 2^–ΔCt^ method (46). Water instead of cDNA was used as a negative control for qPCR experiments. TaqMan probes used in this study are listed in Supplemental Table 1.

### Single-molecule RNA in situ hybridization.

After fixation, adrenal glands were embedded in paraffin blocks and sectioned at 5 μm thickness. Single-molecule RNA in situ hybridization was performed using a RNAscope 2.5 HD Brown Reagent Kit (Advanced Cell Diagnostics, 322300), following the manufacturer’s protocol. Target retrieval was performed for 7 min (33). Slides were counter-stained with 30% Gill’s Hematoxylin (Fisher Scientific, 23-245654) and mounted with Cytoseal XYL (Fisher Scientific, 22-050-262). The following probe was used: *Cyp11b2* (ACD, 505851).

### Serum corticosterone measurements.

Blood was collected from the orbital sinus of 10-week-old male control and Fgfr2-cKO mice, and sera were stored at –80°C. Corticosterone levels were measured using the Corticosterone ELISA kit (ALPCO, 55-CORMS-E01), following the manufacturer’s protocol.

### Metabolic cage experiments.

To assess aldosterone secretion across selected genotypes, Control (*AS^Cre/+^* *R26R^mTmG/+^*), βCat-GOF (*AS^Cre/+^* *Ctnnb1^fl(ex3)/+^* *R26R^mTmG/+^*), and βCat-GOF Fgfr2-cKO (*AS^Cre/+^* *Ctnnb1^fl(ex3)/+^ Fgfr2^fl/fl^* *R26R^mTmG/+^*) adult female mice (6–20 weeks of age) were used, housed in a temperature and humidity controlled environment on a 12:12 light:dark cycle. Metabolic cage experiments were conducted as described previously (47). Mice were individually housed in metabolic cages designed for urine collection, with ad libitum access to water and chow. Mice were fed standard rodent chow for 4 days, followed by low-sodium chow for 7 days. Urine was collected daily. Urine measurements in the first 4 days (normal chow), and the last 4 days (low-sodium chow) were averaged.

To assess aldosterone secretion in WT mice and βCat-GOF mice treated with FGFR inhibitors, adult male C57BL/6J mice ~12 weeks of age and adult female βCat-GOF mice (36–39 weeks of age) were housed, as above. In addition, mice were given a ~100 mg pellet of peanut butter (Jif creamy peanut butter, Kroger) on their drinking spout daily; by the second day, all the mice consumed the peanut butter within 2 minutes. After a habituation period of 4 days, urine was sampled every 24 hours for 10 days: 4 days of baseline measurements and 6 days of drug/vehicle. During the last 6 days, mice received either a plain peanut butter pellet or a peanut butter pellet containing the FGFR inhibitor AZD4547 (19) or BGJ398 (23) (both at 30 mg/kg/day). Peanut butter/drug suspension was formulated as previously described (48).

### Urine and plasma hormone analysis.

Urinary aldosterone was measured using an aldosterone ^125^I radioimmunoassay (Tecan, Morrisville, NC), and standardized to urinary creatinine (Jaffe’s colorimetric assay, Cayman Chemicals, Ann Arbor, MI) to account for differences in glomerular filtration rate and urine volume.

### Calcium imaging in adrenal slices.

Calcium imaging and data analysis followed a previously established protocol (18). Adrenal slices approximately 70 μM thick were prepared from FGFR2-cKO and control mice (AS^Cre/+^) expressing Cre-dependent GCaMP6. Images were acquired at 10 Hz using a Zeiss Axio-Examiner microscope, employing a 63x dipping objective, X-Cite XLED, and Slidebook 6 software (3i). To determine the number of active cells following Ang II stimulation, a 10x dipping objective was utilized, enabling the inclusion of a broader section of the slice.

Imaging was performed under continuous perfusion with PIPES buffer, which comprised 20 mmol/L PIPES, 119 mmol/L NaCl, 4 mmol/L KCl, 2 mmol/L CaCl2, 1 mmol/L MgCl2, 25 mmol/L D-Glucose, and 0.1% BSA. The pH of this buffer was adjusted to 7.3 using 10N NaOH. Following a baseline acquisition period of 1.5 minutes, 300 pM Ang II (Bachem, H-1705) was added to the bath perfusion for the remainder of the experiment, which lasted at least 10 minutes.

Regions of interest (ROIs) representing activity within an individual zG cell were identified using Mesmerize software, an open-source calcium imaging toolbox. Fluorescent intensity over time was plotted for each ROI within a slice, and calcium events determined using custom MATLAB software.

### Statistics.

All statistical analyses were performed and graphs prepared using Prism 10 (GraphPad). Two-tailed Student’s *t* test was used for comparisons between 2 groups. Calcium bursting activity was analyzed using a 2-level nested model. The first level treats each slice as an independent replicate (*N*, 1 slice/mouse). At the second level, individual cells (pseudo-replicates) are nested within these slices. Significance is determined using a nested *t* test with *P* < 0.05. One-way ANOVA with Tukey’s post hoc analysis or 2-way ANOVA with Fisher’s least significant difference post hoc analysis were used for comparisons between groups of 3 or more. The statistical details of the experiments can be found in the figure legends, whereby *n* values correspond to the number of independent mice. Data are presented as mean ± SEM.

### Study approval.

All animal procedures were approved by the Institutional Animal Care and Use Committees of both Boston Children’s Hospital and the University of Virginia.

### Data availability.

All data are available from the corresponding author and are provided in the Supporting Data Values file.

## Author contributions

VC, DK, MB, SL, NAG, PQB, and DTB conceptualized the study and developed the methodology; VC, DK, MB, SL, CR, NAG, BH, MSS, EP, BJ, LG, KSB performed the experiments; all authors interpreted the data; VC, DLC, MB, and DTB wrote the original paper with editorial inputs from all authors; and PQB and DTB provided the supervision and acquired the funds. Authorship order among first coauthors was agreed to based on the time each author worked on the project.

## Funding support

This work is the result of NIH funding, in whole or in part, and is subject to the NIH Public Access Policy. Through acceptance of this federal funding, the NIH has been given a right to make the work publicly available in PubMed Central.

- AHA 18TPA34230077 and R01 DK123694 to DTB
- Michail Papamichail, MD, PhD, Postdoctoral Fellowship at Harvard Medical School to VC.

## Supplementary Material

Supplemental data

Supporting data values

## Version 1 09/16/2025

In-Press Preview

## Version 2 11/10/2025

Electronic publication
