# Supplementary material for: Abrogation of FGFR signaling blocks β-catenin–induced adrenocortical hyperplasia and aldosterone production
Source: JCI Insight. 2025 Sep 16;10(21):e184863. doi: 10.1172/jci.insight.184863 (PMC12643481; doi:10.1172/jci.insight.184863)
Supplement: Supplemental data [file jciinsight-10-184863-s129.pdf]

Supplemental Figure 1

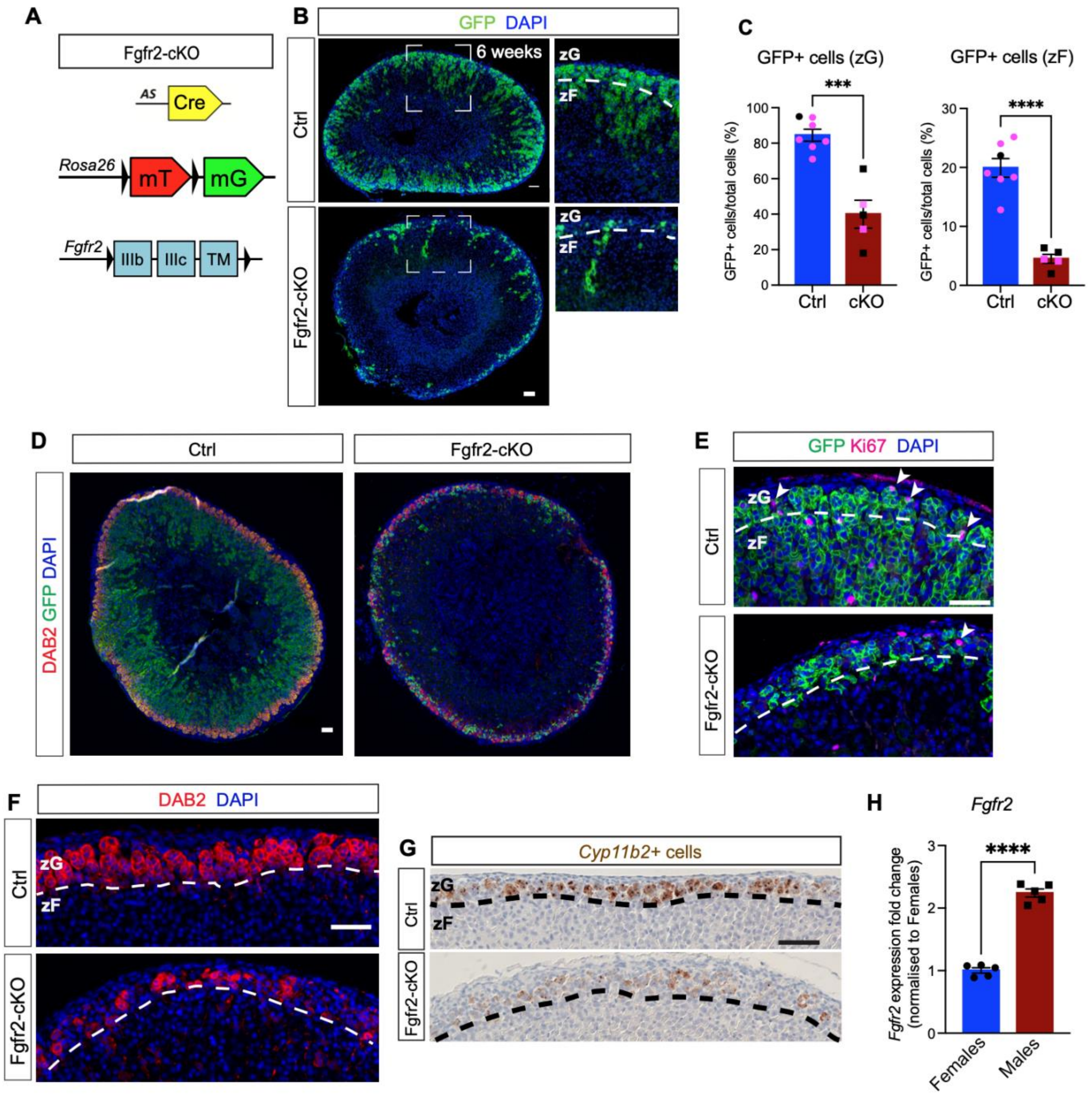

Supplemental Figure 1 (Supplemental to Figure 1): Constitutive *Fgfr2* knock-out (Fgfr2-cKO) impairs zG cell identity and transdifferentiation. (**A**) Schematic of the Fgfr2-cKO ($AS^{Cre/+}$::$Fgfr2^{fl/fl}$::$R26R^{mTmG/+}$) model. (**B**) Lineage-tracing by immunofluorescence for GFP+ cells in adrenals from 6-week old female Fgfr2-cKO and control (Ctrl, $AS^{Cre/+}$::$R26R^{mTmG/+}$) mice. White dashed box demarcates region enlarged to the right. (**C**) Quantification of GFP+ cells as a proportion of total adrenocortical cells in the zG and zF from 6-10-week-old male and female control (Ctrl) and Fgfr2-cKO (cKO) mice. Adrenals from male mice are represented with black dots; adrenals from female mice are represented with magenta dots. Student's t-test, *** $p < 0.001$, **** $p<0.0001$, N=7, 5 mice. (**D**) Representative lineage-tracing by immunofluorescence for GFP+ cells (green) and co-staining with DAB2 (red) in adrenals from adult 12-week-old male Ctrl and Fgfr2-cKO mice. (**E**) Representative images of GFP (Green) and Ki67 (Magenta) immunostaining in adrenals from adult 12-week-old male Ctrl and Fgfr2-cKO mice. White arrowheads point to GFP and Ki67 co-positive cells. (**F**) Representative images of DAB2 (red) immunostaining in adrenals from adult male Fgfr2-cKO and Ctrl mice. (**G**) Representative bright-field images of *in situ* hybridization (RNAscope) of *Cyp11b2* (brown) in adrenals from adult male mice. Nuclei are stained with hematoxylin. (**H**) *Fgfr2* expression (qRT-PCR) in male and female adrenals from wild-type adult C57BL/6 mice. Student's t-test, **** $p<0.0001$, N=5, 5 mice. All immunostaining: Scale Bars, 50µm. DAPI (blue), nuclei. Dashed lines correspond to the zG-zF boundary.

Supplemental Figure 2

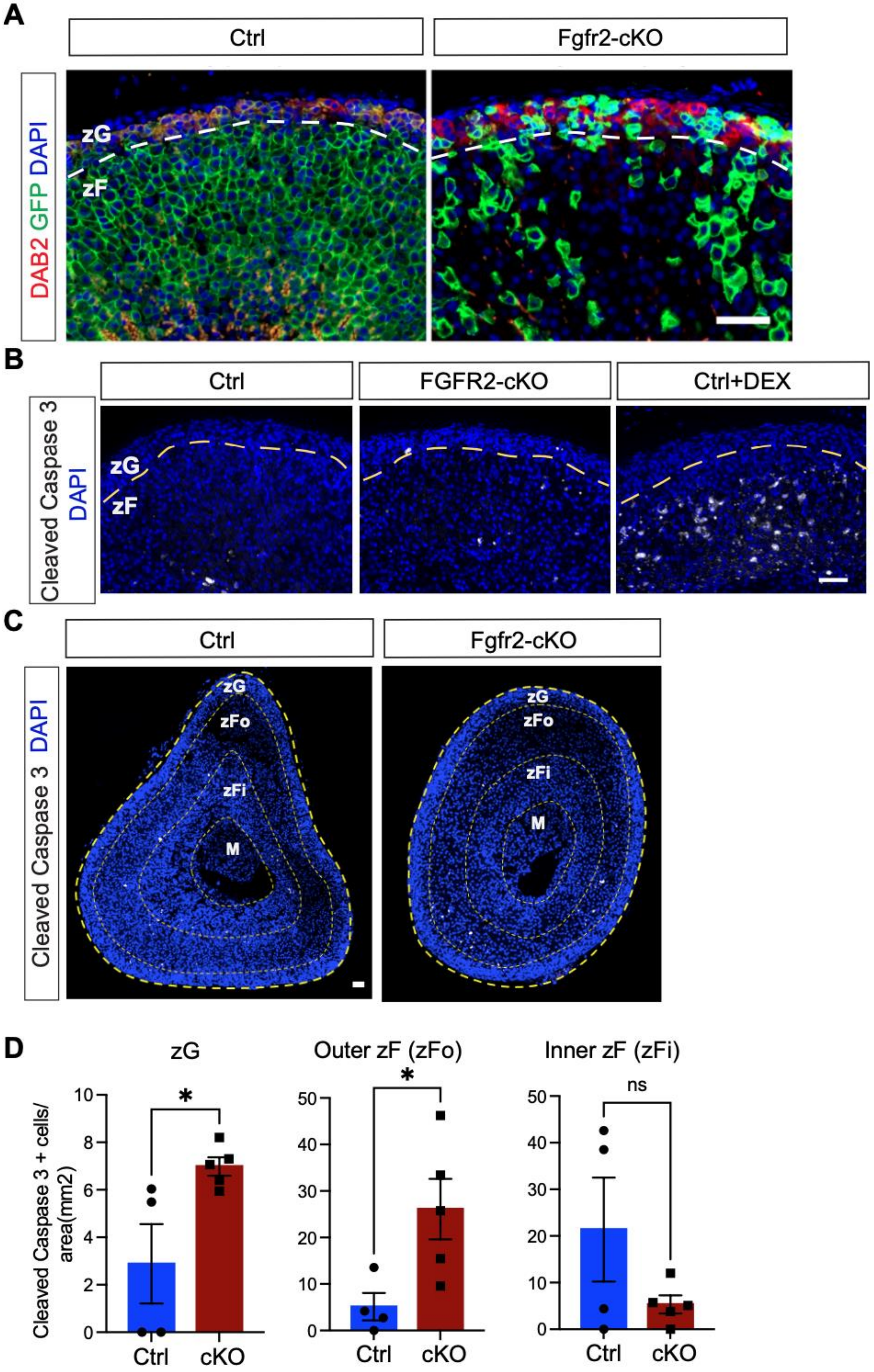

Supplemental Figure 2 (Supplemental to Figure 1): Apoptosis assessment in Fgfr2-cKO mice and adrenal morphology in aged animals. (**A**) Representative images of GFP (Green) and DAB2 (Red) immunostaining in adrenals from aged Ctrl (8 months) and Fgfr2-cKO (18-20 months) female mice. Dashed lines correspond to the zG-zF boundary. (**B**) Representative images of cleaved caspase 3 immunostaining (grey) in adrenals from Ctrl and Fgfr2-cKO male mice (14-18 weeks), and dexamethasone-treated male mice shown as a positive control. Dashed line corresponds to the zG-zF boundary. (**C,D**) Representative images (**C**) and spatial quantification (**D**) of cleaved Caspase 3+ cells in whole adrenals from Ctrl and Fgfr2-cKO female mice. Dashed lines define borders of the zG, outer zF (zFo), inner zF (zFi), and medulla (M). Student's t-test, *$p<0.05$, ns: not significant, N=4,5 mice. All immunostaining: Scale Bars, 50µm. DAPI (blue), nuclei.

.

Supplemental Figure 3

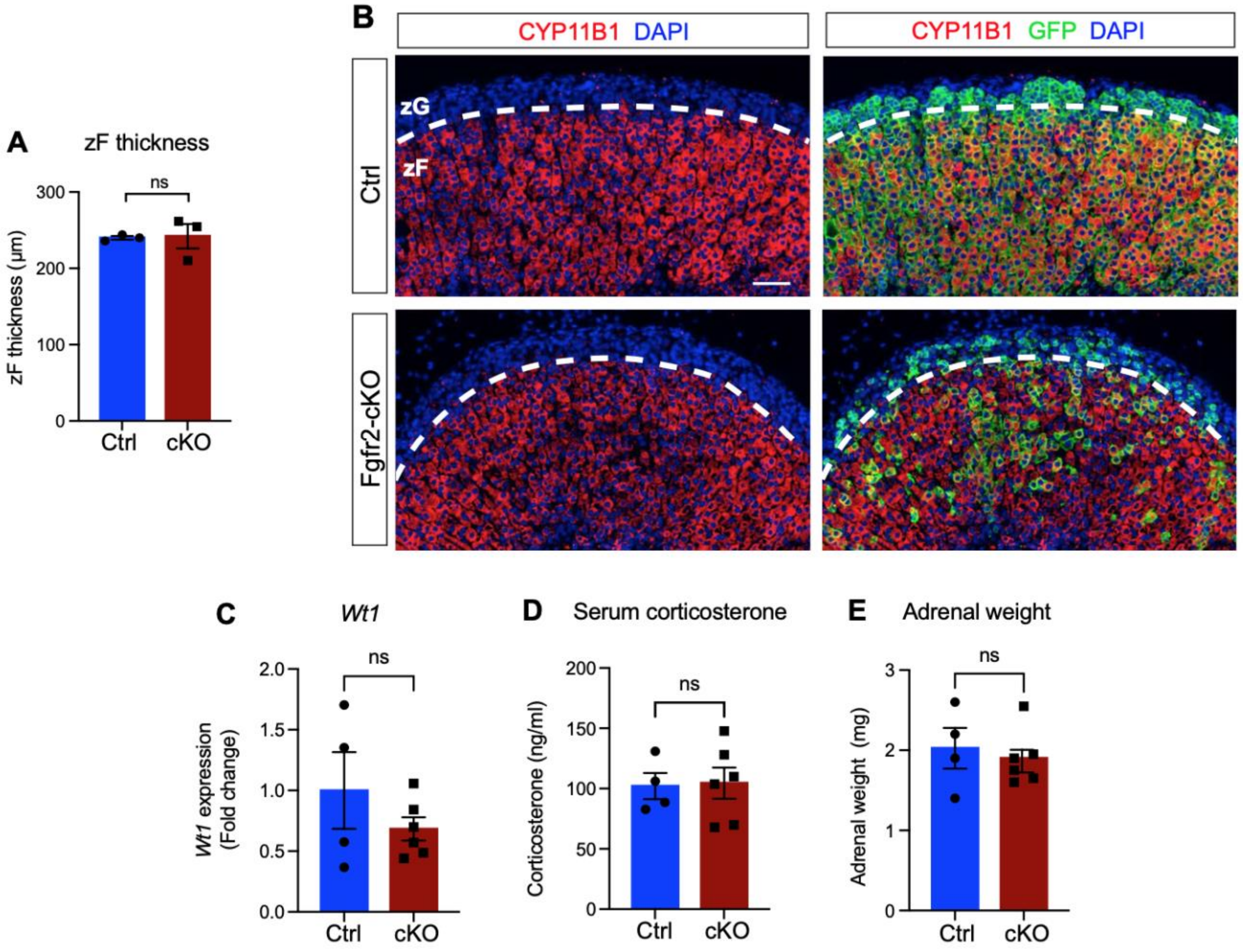

Supplemental Figure 3 (Supplemental to Figure 1): zF morphology and function in Fgfr2-cKO mice. (**A**) zF thickness in adrenals from female control (Ctrl) and Fgfr2-cKO (cKO) mice (20-24 weeks). Student's t-test, ns: not significant, N=3, 3 mice. (**B**) Representative images of GFP (green) and CYP11B1 (red) co-immunostaining of control (Ctrl) and Fgfr2-cKO (cKO) adrenals from adult female mice. Scale Bar, 50μm. DAPI (blue), nuclei. White dashed line corresponds to the zG-zF boundary. (**C**) mRNA expression (qRT-PCR) levels of *Wt1* (Wilms Tumor suppressor gene 1), assessed in adrenals from male Ctrl and Fgfr2-cKO (cKO) mice (12 weeks). Student's t-test, ns: not significant, N=4, 6 mice (**D**) Serum corticosterone levels in male Ctrl and Fgfr2-cKO (cKO) mice (12 weeks). Student's t-test, ns: not significant, N=4, 6 mice. (**E**) Adrenal weight in male Ctrl and Fgfr2-cKO (cKO) mice (12 weeks). Student's t-test, ns: not significant, N=4, 6 mice

Supplemental Figure 4

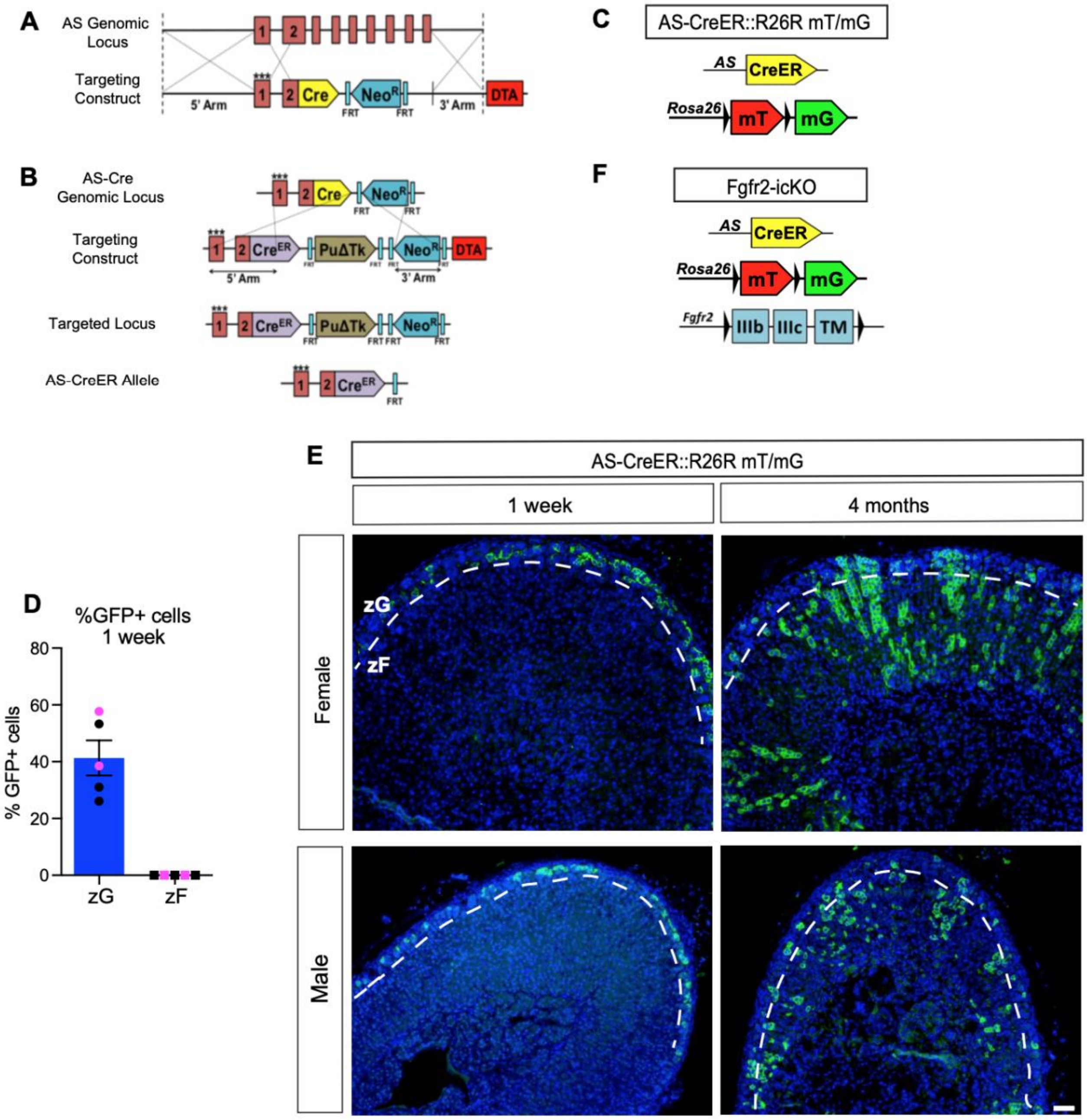

Supplemental Figure 4 (Supplemental to Figure 2): Introduction and characterisation of the tamoxifen-inducible AS-Cre mouse model (AS-CreER). (**A**) Schematic showing the endogenous AS genomic locus and the targeting construct employed to generate the original AS^{Cre} mice with 5' and 3' homologous targeting arms, a FRT-flanked neomycin resistance cassette (NeoR) for positive selection and a Diphtheria toxin subunit A (DTA) cassette for negative selection. All translation start sites (ATG) within exon 1 or 2 were excluded from the construct or mutated, as indicated by the three asterisks above exon 1. (**B**) Schematic showing the targeting strategy used to generate tamoxifen-inducible AS^{CreER} mice by retargeting the AS^{Cre} locus in the embryonic stem cell line used to generate AS^{Cre} mice. The targeting construct consists of a 5' homology arm containing exon 1 and part of exon 2 of the *AS* allele followed by the Cre^{ERT2} coding sequence (denoted in the schematic as Cre^{ER}), a FRT-flanked puromycin positive selection cassette (PuΔTk) and a 3' homology arm containing an FRT-flanked neomycin resistance cassette as well as and a Diphtheria toxin subunit A (DTA) cassette for negative selection. (**C**) Schematic of tamoxifen-inducible $AS^{CreER/+}$::$R26R^{mTmG/+}$ model (AS-CreER::R26R mT/mG). (**D**) Fractional GFP expression in the zG and zF of AS-CreER::R26R mT/mG mice 1 week post tamoxifen induction. Adrenals from male mice are represented with black dots; adrenals from female mice are represented with magenta dots. (**E**) Representative images of lineage-tracing by immunofluorescence for GFP+ cells (green) in adrenals from adult male and female AS-CreER::R26R mT/mG mice one week and four months post tamoxifen administration (tamoxifen administered at six weeks of age). Scale Bar, 50μm. DAPI (blue), nuclei. White dashed line corresponds to the zG-zF boundary. (**F**) Schematic of the tamoxifen-inducible Fgfr2-icKO ($AS^{CreER/+}$::$Fgfr2^{fl/fl}$::$R26R^{mTmG/+}$) model.

Supplemental Figure 5

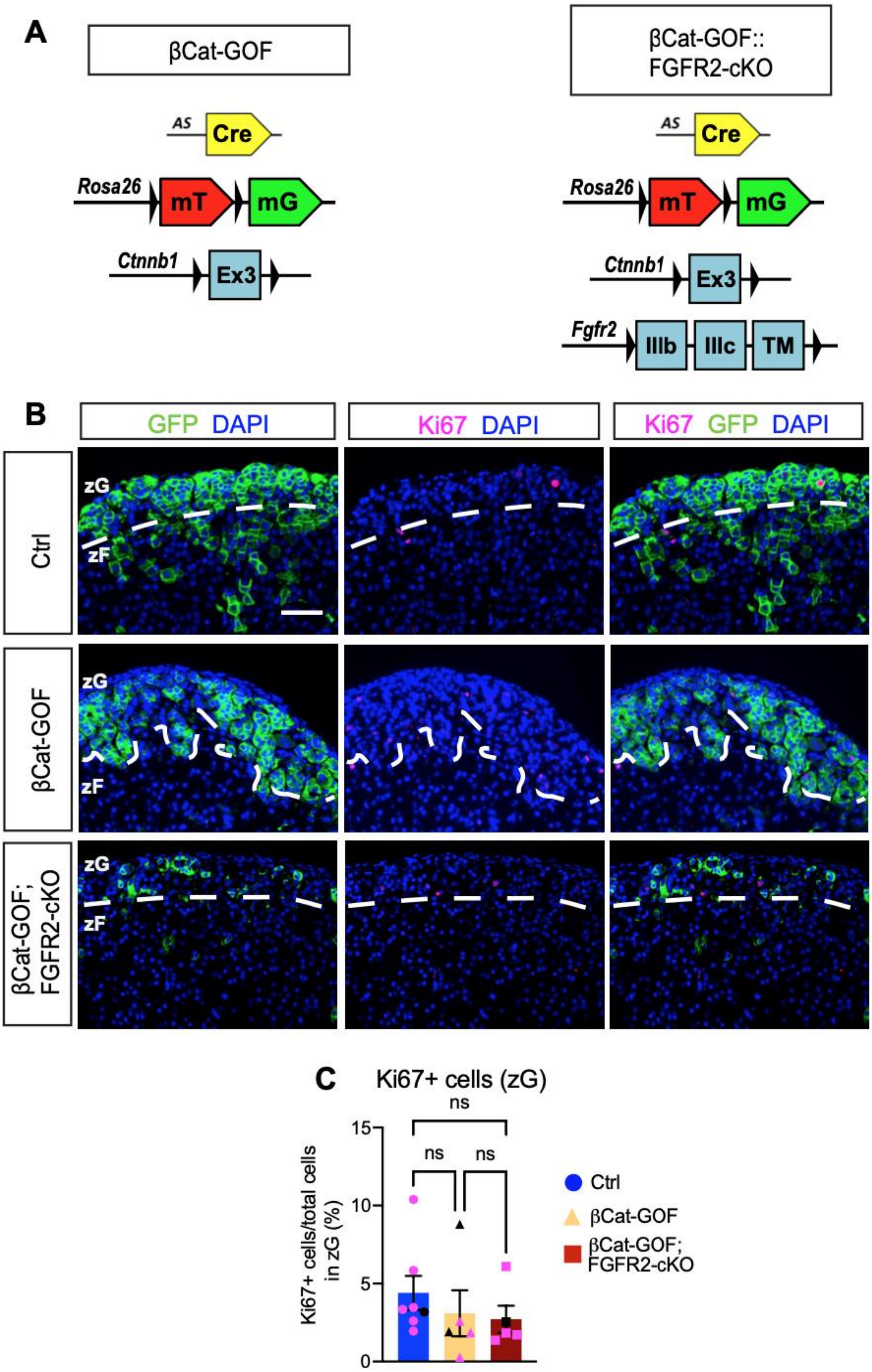

Supplemental Figure 5 (Supplemental to Figure 3): zG cell proliferation is unchanged in Control, βCat-GOF and βCat-GOF; Fgfr2-cKO mice. (**A**) Schematic of the βCat-GOF ($AS^{Cre/+}$::$R26R^{mTmG/+}$::$Ctnnb1^{ex3/+}$), and βCat-GOF::Fgfr2-cKO ($AS^{Cre/+}$:: $R26R^{mTmG/+}$::$Ctnnb1^{ex3/+}$::$Fgfr2^{fl/fl}$) models. (**B**) Representative images of GFP (green) and Ki67 (magenta) immunostaining of control (Ctrl, $AS^{Cre/+}$::$R26R^{mTmG}$), βCat-GOF and βCat-GOF::Fgfr2-cKO adrenals (adult mice aged 6-10 weeks). Scale Bar, 50µm. DAPI (blue), nuclei. White dashed line corresponds to the zG-zF boundary. (**C**) Quantification of Ki67+ cells in the zG from male and female Ctrl, βCat-GOF and βCat-GOF::Fgfr2-cKO mice. Adrenals from male mice are represented with black data points; adrenals from female mice are represented with magenta data points. One-way ANOVA, P=0.56, ns: not significant.

Supplemental Figure 6

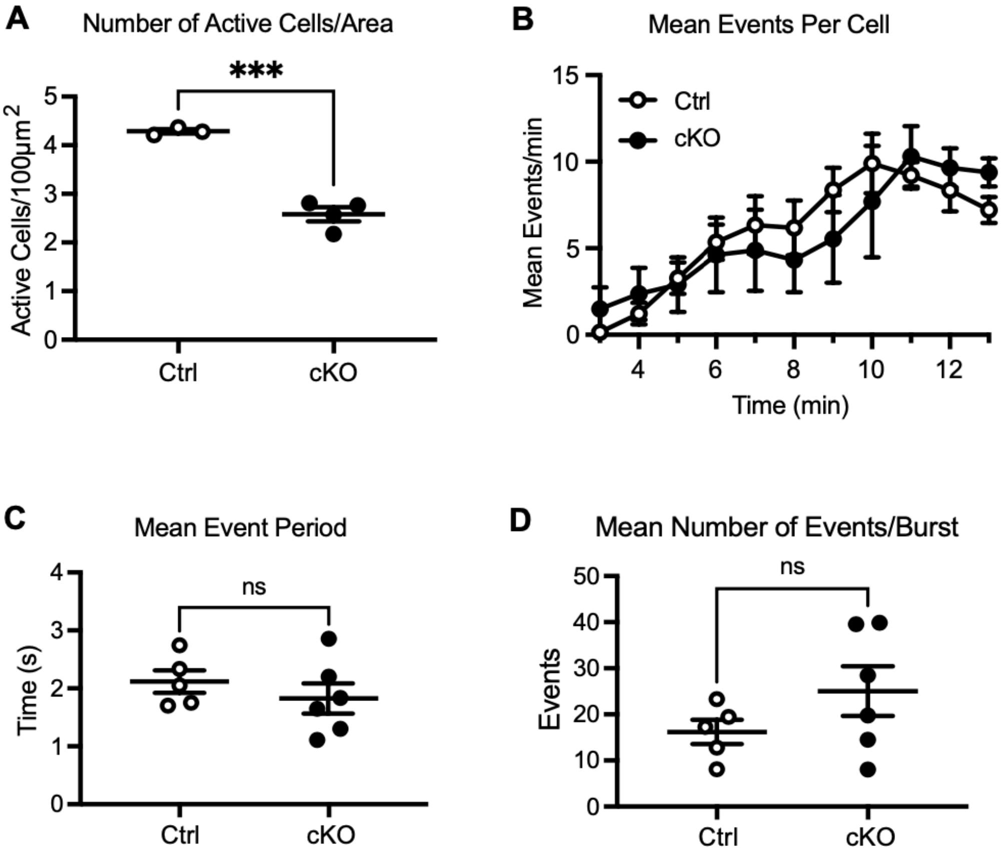

Supplemental Figure 6: Calcium imaging in Fgfr2-cKO adrenals using the GCaMP6 calcium reporter system. (**A**) Number of active cells per 100 μM^2 of zG in Ctrl and Fgfr2-cKO adrenal slices from 7-12 weeks old mice, recorded from 3-11 min after stimulation with 300 pM Ang II. Student's t-test, ***$p<0.001$, N=5, 4 per group. (**B**) Mean number of events (calcium oscillations) elicited by 300pM Ang II added to the media at 1.5 minutes per active cell binned in 1-minute intervals. (**C**) Mean event period and (**D**) mean number of events/calcium burst. (**B-D**) Data presented as mean of active cells analysed for each mouse (circle). Nested t-test, N=5, 6.

Supplemental Figure 7

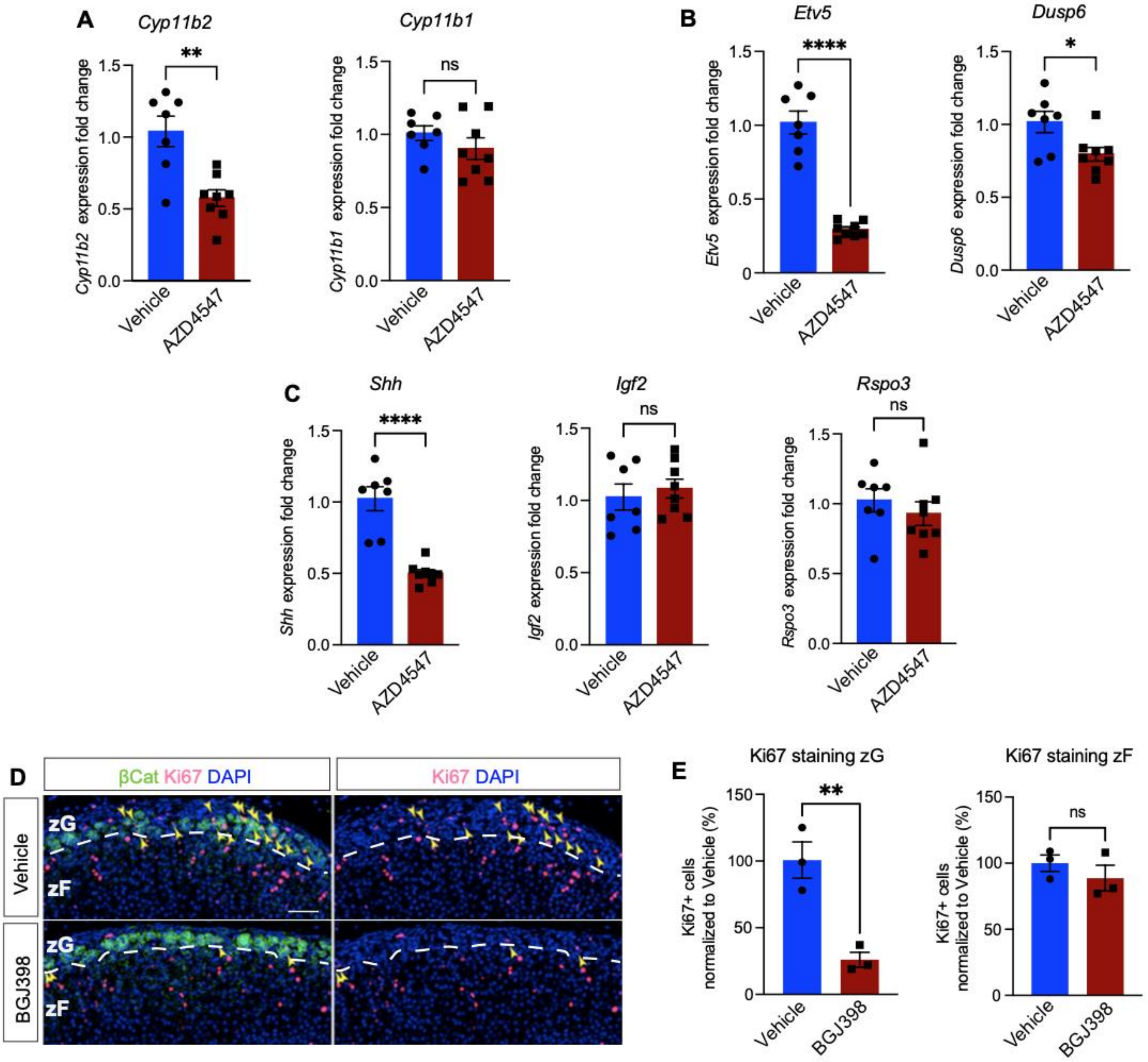

Supplemental Figure 7 (Supplemental to Figure 4): Pharmacological FGFR inhibition impedes zG cell proliferation. (A-B) mRNA expression levels (qRT-PCR) of zG and zF markers *Cyp11b2* and *Cyp11b1*, respectively (A) and downstream FGFR2 targets *Etv5* and *Dusp6* (B) in adrenals from male C57BL/6J mice (7-8 weeks) treated with 10 mg/kg/day AZD4547 or vehicle. Student's t-test, N=7, 8 mice**.** (**C**) mRNA expression (qRT-PCR) levels of *Shh, Rspo3* and *Igf2* in adrenals from adult male C57BL/6J mice (7-8 weeks) treated with 10 mg/kg/day of AZD4547 or vehicle by oral gavage. Student's t-test: ****p<0.0001, ns: not significant, N=7,8. (**D**) Representative images of Ki67 (magenta) and β-Catenin (a zG marker, green) immunostaining in male C57BL/6J male mice (12 weeks) treated with 10 mg/kg/day BGJ398 or vehicle (Veh) for 12 days by oral gavage. Yellow arrowheads indicate Ki67+ cells in the zG. Scale Bars, 50µm. DAPI (blue), nuclei. White dashed line corresponds to the zG-zF boundary. (**E**) Quantification of Ki67+ cells in the zG and the zF of BGJ398 and vehicle-treated mice. Student's t-test, ** p < 0.01, ns: not significant, N=3, 3 mice.

Supplemental Table 1. Taqman probes used for qRT-PCR

**Gene Name**	**Taqman Probe**
Actb	Mm02619580
Cyp11b1	Mm01204952_m1
Cyp11b2	Mm01204955_g1
Etv5	Mm00465816_m1
Dusp6	Mm00518185_m1
Hprt	Mm03024075_m1
Igf2	Mm00439564_m1
Ren	Mm02342887_mH
Rspo3	Mm01188251_m1
Shh	Mm0436528_m1
Wt1	Mm01337048
